# Comparison of carcinoembryonic antigen prognostic value in serum and tumour tissue of patients with colorectal cancer

**DOI:** 10.1111/j.1463-1318.2008.01591.x

**Published:** 2009-03

**Authors:** M Li, J-Y Li, A-L Zhao, J-S He, L-X Zhou, Y-A Li, J Gu

**Affiliations:** * Gastrointestinal Surgery Unit, Peking University School of Oncology, Beijing Cancer Hospital & InstituteBeijing, China; † Department of Pathology, Peking University School of Oncology, Beijing Cancer Hospital & InstituteBeijing, China

**Keywords:** Colorectal cancer, prognosis, carcinoembryonic antigen

## Abstract

**Objective:**

Carcinoembryonic antigen (CEA) in the serum and the tumour tissue of colorectal cancer (CRC) patients is the most commonly used tumour marker for the diagnosis and evaluation of prognosis or recurrence after treatment, but the role remains controversial. The objective of this study was to compare the prognostic value of CEA both in serum and tumour tissue in CRC.

**Method:**

A total of 173 patients with CRC in stages I–III were retrospectively assessed with the endpoint of recurrence or metastasis after curative operation. CEA was assessed both in serum and tumour tissue.

**Results:**

37.0% (64/173) patients had a high level of CEA in serum (S-CEA) while 39.3% (68/173) had high CEA in tumour tissue (T-CEA). There were no significant differences in clinico-pathological features between the low and high S-CEA or T-CEA groups. The high S-CEA group had a worse prognosis than the low S-CEA group but the difference was not significant. The high T-CEA group had a significantly poorer prognosis than the low T-CEA group (*P*=0.028) in the univariate analysis. The multivariate analysis demonstrated that the T-CEA was an independent prognosis factor in CRC. Because many factors would affect the concentration of S-CEA, there was no correlation between S-CEA and T-CEA directly.

**Conclusion:**

Our study suggests that a high T-CEA concentration may be a useful and independent predictor for poor outcome after surgery in CRC patients. It may be stronger than a high preoperative serum CEA level.

## Introduction

Colorectal cancer (CRC) is one of the most common tumour types in the world, with about 400 000 deaths annually [1,2]. In the United States, despite a slight decrease in its incidence and mortality during the past two decades, CRC remains the third most common cancer, affecting about 140 000 people and accounting for 50 000 cancer-related deaths per year [3]. In China, where the incidence rate was initially low, due to the changes in lifestyle and nutritional habits, the CRC rate is increasing by 4.2% annually [4,5].

Carcinoembryonic antigen (CEA) is the most commonly used tumour marker for the diagnosis of CRC and evaluation of prognosis or recurrence after treatment. The guideline of National Comprehensive Cancer Network (NCCN) indicated that for T2 or greater lesions, a CEA test is recommended at baseline and every 3 months for 2 years [6]. CEA can be detected and quantitatively measured in the serum and the tumour tissue of CRC patients, but their role in the prognosis of CRC remains controversial. The objective of this study was to compare the prognostic value of CEA both in tumour tissue and in serum of the patients with CRC.

## Method

### Patients

This retrospective study included 173 patients from the database of Beijing Cancer Hospital between January 1995 and November 1999 who satisfied the following criteria: (1) sporadic CRC diagnosed in our hospital; (2) no preoperative therapy; (3) curative resection with free margin; (4) no synchronic liver or other organ metastasis detected; (5) followed up until patients had metastasis or local recurrence or until the last census point prior to closure of the study (1 August 2003); (6) staged according to the TNM system recommended by the American Joint Committee on Cancer (AJCC) [7].

The criterions for exclusion were: (1) severe dysfunction of heart, brain, lung, kidney and liver; (2) death attributable to causes other than CRC; (3) accompanying urological or genital tumour; (4) accompanying cancers other than CRC.

### Detection of the CEA in serum

Five millilitres of venous blood was obtained from each patient 1 week before operation. CEA in serum (S-CEA) measurements were done by the department of clinical laboratory in our hospital using electrochemiluminescence immunoassay with Elecsys system 2010 (Roche Holding Ltd, Basel, Switzerland). The cut-off value of S-CEA recommended by the manufacturers for diagnosis was 5 ng/ml. We classified the 173 patients into high S-CEA group (> 5 ng/ml) and low S-CEA group (≤ 5 ng/ml).

### Immunohistochemistry

The CEA in the tumour tissue which we named T-CEA was determined by the method of immunohistochemistry. In each patient, formalin-fixed and paraffin-embedded tumour blocks were cut into 5 μm thick sections (average area 2.0 cm^2^), deparaffinized in xylene, and rehydrated. Antigen retrieval was performed in 0.1 m citric acid buffer (pH 6.0) in a 650 W microwave for 15 min. Endogenous peroxidase activity was blocked with 0.3% hydrogen peroxide in 100% methanol for 30 min. Immunohistochemistry was performed as Power Vision™ two-step histostaining (Immuno Vision Technologies Co., Daly City, CA, USA). Primary CEA antibody (Zymed Laboratories Inc., South San Francisco, CA, USA) was diluted as 1:50 and sections were incubated overnight at 4°C. After three washes in PBS (5 min), sections were incubated for 30 min in anti-mouse secondary antibody from a PicTure™-PV6000 Kit (Zymed Laboratories Inc.). Antibody binding was visualized using a 3,3-diaminobenzidine (DAB) kit (Vector Labs, Burlingame, CA, USA) according to the manufacturer’s instructions.

### Scoring

For general negative controls, the primary antibodies were replaced by phosphate buffer solution (PBS). All slides were scored by two independent and well-trained pathologists, without the knowledge of clinical and pathologic parameters or the patients’ outcomes. And in cases of scoring disagreement, a third independent assessment was performed. For all slides, at least 10 high power fields at 400× magnification were chosen randomly and > 1000 carcinoma cells were counted for each section.

**Figure 1:**
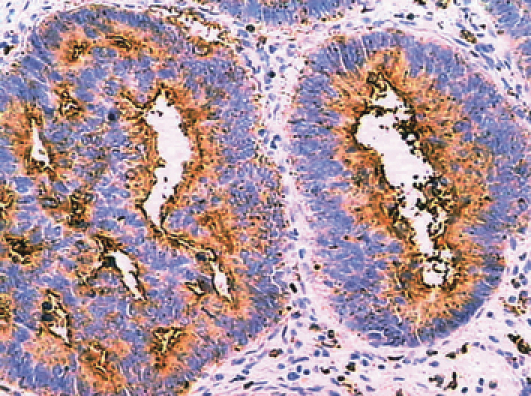
High expression of CEA in colorectal cancer (≥ 75%).

Stained slides were examined to identify the cellular localization of CEA immunoreactivity for both intensity (−, +, ++, and +++) and proportion (0%, 1–5%, 6–25%, 26–50%, 51–75% and > 75%) of tumour cells stained. Integer values were assigned to the scores of intensity (0–3) and proportion of tumour cells stained (0–5). These values were multiplied together to provide a single score for each case [8]. For T-CEA, the low and high expression scores were defined as < 6 and ≥ 6, respectively (Fig. 1) [9].

### Statistical analysis

All statistical calculations were performed using spss version 10.0 (SPSS Inc., Chicago, Illinois, USA). The differences in the clinico-pathological characteristics between low and high S-CEA or T-CEA expression were performed by Pearson chi-square. The relationship between S-CEA and T-CEA was also calculated by Pearson chi-square. Disease-free survival time (in months) was measured from the date of surgery to the time of event (recurrence or metastasis) or to the last census prior to closure of the study (1 August 2003). Kaplan–Meier survival analysis with the Log Rank test was used to evaluate the prognosis of serum and tissue CEA in CRC. Multivariate analysis was performed with Cox’s proportional hazard regression model to assess the effects of different variables on patients’ survival. Differences were taken as significant when *P* (two-tailed) was < 0.05.

## Results

In this study, there were 173 patients with CRC including 86 male subjects and 87 female subjects. The median age of all patients was 59 years (range 27–85 years). 37.0% (64/173) patients had a high level of S-CEA including 29 male subjects and 35 female subjects, while 63.0% (109/173) patients were in the low S-CEA group. 39.3% (68/173) patients were in the high T-CEA group (38 male subjects and 30 female subjects).

### Comparison of clinico-pathological features between high and low S-CEA or T-CEA

There were no significant differences in gender, age, tumour size, tumour gross type, mucin production, differentiation grade, venous invasion, stage distribution, T and N classification between the low and high S-CEA or T-CEA groups (Table 1).

**Table 1:** Clinico-pathological characteristics in S-CEA and T-CEA group *n* (%).

	Total (*n* = 173)	Low S-CEA (*n* = 109)	High S-CEA (*n* = 64)	*P-*value	Low T-CEA (*n* = 105)	High T-CEA (*n* = 68)	*P-*value
Gender							
Male	86 (49.7)	57 (52.3)	29 (45.3)	0.375	48 (45.7)	38 (55.9)	0.191
Female	87 (50.3)	52 (47.7)	35 (54.7)		57 (54.3)	30 (44.1)	
Age							
Median	59.0	56.4	58.1	0.408	61	56.50	0.603
Range	27–85	33–85	27–82		27–85	30–76	
Tumour size							
≤ 5 cm	78 (45.1)	48 (44.0)	30 (46.9)	0.844	45 (42.9)	33 (48.5)	0.657
> 5 cm	91 (52.6)	58 (53.2)	33 (51.6)		58 (55.2)	33 (48.5)	
Unknown	4 (2.3)	3 (2.8)	1 (1.6)		2 (1.9)	2 (3.0)	
Gross type							
Ulcerative	131 (75.7)	82 (75.2)	49 (76.6)	0.843	77 (73.3)	54 (79.4)	0.362
Polypoid	42 (24.3)	27 (24.8)	15 (23.4)		28 (26.7)	14 (20.6)	
Mucin production							
Mucinous adenocarcinoma	13 (7.5)	6 (5.5)	7 (10.9)	0.191	9 (8.6)	4 (5.9)	0.512
Adenocarcinoma	160 (92.5)	103 (94.5)	57 (89.1)		96 (91.4)	64 (94.1)	
Differentiation grade							
Undifferentiated	7 (4.1)	5 (4.6)	2 (3.1)	0.871	6 (5.7)	1 (1.5)	0.318
Poorly	31 (17.9)	21 (19.3)	10 (15.6)		21 (20)	10 (14.7)	
Moderate	90 (52.0)	56 (51.4)	34 (53.1)		50 (47.6)	40 (58.8)	
Well	45 (26.0)	27 (24.8)	18 (28.1)		28 (26.7)	17 (25)	
Venous invasion							
No	140 (77.8)	89 (81.7)	51 (79.7)	0.751	86 (80.4)	54 (75.8)	0.684
Yes	33 (22.2)	20 (18.3)	13 (20.3)		19 (19.6)	14 (24.2)	
Stage							
I	22 (11.3)	17 (15.6)	5 (7.8)	0.179	16 (5.8)	6 (15.6)	0.130
II	75 (40)	49 (45.0)	26 (40.6)		49 (47.1)	26 (34.4)	
III	76 (48.7)	43 (39.4)	33 (51.6)		40 (47.1)	36 (50)	
T							
T1	2 (1.1)	2 (1.8)	0 (0)	0.517	2 (1.9)	0 (0)	0.392
T2	27 (15.6)	19 (17.4)	8 (12.5)		18 (17.1)	9 (13.2)	
T3	106 (61.3)	66 (60.6)	40 (62.5)		60 (57.2)	46 (67.7)	
T4	38 (22)	22 (20.1)	16 (25)		25 (23.8)	13 (19.1)	
N							
N0	97 (56.1)	66 (60.6)	31 (48.4)	0.099	65 (61.9)	32 (47.1)	0.099
N1	40 (23.1)	24 (22.0)	16 (25)		20 (19.1)	20 (29.4)	
N2	30 (17.3)	17 (15.6)	13 (20.3)		15 (14.2)	15 (22.0)	
N3	6 (3.5)	2 (1.8)	4 (6.3)		5 (4.8)	1 (1.5)	

### The relationship between S-CEA and T-CEA groups

There was no significant relationship between groups of S-CEA and T-CEA (*P*=0.215) (Table 2).

**Table 2:** The relationship between S-CEA and T-CEA groups.

	Low T-CEA (*n* = 105)	High T-CEA (*n* = 68)	*P-*value
Low S-CEA(*n* = 109)	70	39	0.215
High S-CEA(*n* = 64)	35	29	

### Relationship of S-CEA to disease-free survival by univariate analyses

**Figure 2:**
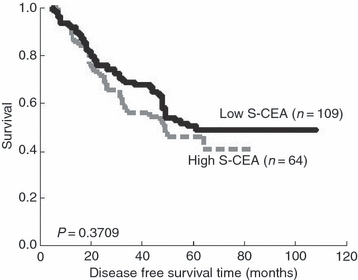
Kaplan–Meier survival analysis for the difference of disease free survival time between low and high S-CEA.

The mean disease-free survival time after operation in the low S-CEA group was longer than patients of high level of S-CEA (68.4 vs 51.3 months, 95% CI), but there was no significant difference between them (*P*=0.3709) (Fig. 2).

### Relationship of T-CEA with disease-free survival by univariate analyses

**Figure 3:**
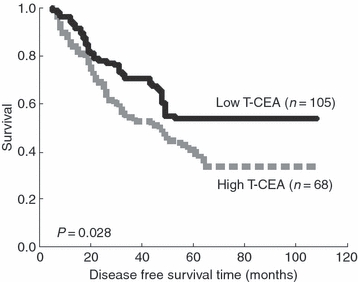
Kaplan–Meier survival analysis for the difference of disease free survival time between low and high T-CEA.

Kaplan–Meier survival analysis with the Log Rank test showed that the mean disease-free survival time after operation in the low T-CEA group was significantly longer than in the high T-CEA group (72.0 *vs* 55.8 months, *P*=0.028) (Fig. 3).

### Multivariate analysis of prognostic factors in colorectal cancer

In order to evaluate which clinico-pathological features were independent predictors of CRC outcomes, we analysed our findings with a Cox proportional hazards model while gender, age, tumour size, histological type, differentiation grade, venous invasion, stage, S-CEA and T-CEA expressions served as covariates. Finally, four independent factors including histological type, stage, venous invasion and T-CEA were found to be significant prognostic factors for the disease-free survival of CRC. S-CEA was not found to be a significant prognostic predictor (Table 3).

**Table 3:** Multivariate analysis of prognostic factors in colorectal cancer.

	Hazard ratio	95% Confidence interval	*P*-value
Mucin adenocarcinoma*	3.416	1.703–6.851	0.001
Stage II†	0.751	0.362–1.558	0.442
Stage III†	1.690	0.809–3.528	0.162
Venous invasion‡	2.028	1.176–3.498	0.011
T-CEA§	1.587	1.037–2.427	0.033

* Reference is adenocarcinoma.

† Reference is stage I.

‡ Reference is no venous invasion.

§ Reference is T-CEA low expression.

## Discussion

Carcinoembryonic antigen was first described in 1965 by Gold and Freedman [10,11], when they identified an antigen that was present in both fetal colon and colon adenocarcinoma but that was absent from the healthy adult colon, hence its name, carcinoembryonic antigen. Subsequent work showed that CEA was also present in certain healthy tissues, although concentrations in tumours were on average 60-fold higher than in the nonmalignant tissues [12]. Now CEA gene is classified as a member of the immunoglobulin supergene family [13,14].

Carcinoembryonic antigen is the most widely used tumour marker worldwide and certainly the most frequently used marker in CRC. It could be detected and measured both in serum and in CRC tissue [15]. The prognostic role of increased CEA level in serum and tumour tissue of CRC patients remains unknown.

In the current study of 173 CRC patients, there was no significant difference in the disease-free survival time between low and high S-CEA. The S-CEA is not an independent prognostic factor for CRC by multivariate analysis. Previous experiments have reported that an elevated preoperative serum CEA level is a predictor for poor survival after CRC resection [16,17], some even suggesting that serum CEA was an independent factor of CRC prognosis [18,19]. By contrast, some studies demonstrated that serum CEA had significant prognostic value only in some special stages or the significance is not independent of staging system, which is similar to our results [20–22]. The present study, along with some previous reports, had revealed no significant relationship between preoperative serum CEA and tumour tissue CEA concentrations [23–27]. The reasons for these inconsistent results may be due to CEA production, release and metabolism. As we know, many factors may affect this course. Firstly, well-differentiated CRCs produce more CEA than poorly differentiated specimens. Similarly, S-CEA tends to be higher in patients with well-differentiated tumours compared with those poorly differentiated tumours [28,29]. Thus, a lack of differentiation or poor differentiation may explain why some patients with advanced CRC do not have increased S-CEA values [30]. Secondly, the liver is the primary site for the metabolism of CEA. Consequently, S-CEA can be increased from patients with impaired liver function such as certain nonmalignant liver diseases [31,32]. Thirdly, some reports suggest that patients with tumours in the left side of the colon generally have a higher incidence of increased S-CEA than those with malignancies on the right side of the colon [33,34]. Fourthly, Sugarbaker [35] showed that bowel obstruction may give rise to S-CEA in patients with CRC and decompression alone can reduce serum CEA values. Fifthly, S-CEA values can be almost doubled by smoking [36]. Finally, patients with aneuploid CRC have been shown to produce higher S-CEA than those with tumours with a near diploid pattern [37]. All these findings make the S-CEA and T-CEA unparallel.

The results of this series suggest that the prognostic value of T-CEA concentration may be superior to that of preoperative S-CEA level. The disease-free survival time after surgery for patients with a high T-CEA concentration was significantly shorter in univariate analysis than those with a low T-CEA. Multivariate analysis also revealed that T-CEA status (high or low) was an independent prognostic factor in CRC. The hazard of recurrence and metastasis postoperatively in the high T-CEA group is 1.587 times compared with the low T-CEA group. It is coincident with the study of Nakagoe *et al.*[38]. We think that the prognostic value of T-CEA concentration may be more reliable than preoperative S-CEA levels. However, strict statistical process in a large number of patients is needed to clarify the issue.

In conclusion, our study suggests that a high T-CEA concentration may be a useful and independent predictor for poor outcome after surgery in CRC patients, and it may be stronger than a high preoperative serum CEA level.
